# Recombinant Destabilase from *Hirudo medicinalis* Is Able to Dissolve Human Blood Clots In Vitro

**DOI:** 10.3390/cimb43030143

**Published:** 2021-11-20

**Authors:** Pavel Bobrovsky, Valentin Manuvera, Izolda Baskova, Svetlana Nemirova, Alexandr Medvedev, Vassili Lazarev

**Affiliations:** 1 Federal Research and Clinical Center of Physical-Chemical Medicine of Federal Medical Biological Agency, 119435 Moscow, Russia; vmanuvera@yandex.ru (V.M.); lazarev@rcpcm.org (V.L.); 2 Moscow Institute of Physics and Technology, 141701 Dolgoprudny, Moscow Region, Russia; 3 Federal State Budget Educational Institution of Higher Education M.V. Lomonosov Moscow State University, 119991 Moscow, Russia; ipbaskova@bk.ru; 4 Federal State Budgetary Educational Institution of Higher Education “Privolzhsky Research Medical University” of the Ministry of Health of the Russian Federation, 603005 Nizhny Novgorod, Russia; nemirova.info@gmail.com (S.N.); medvedev.map@yandex.ru (A.M.)

**Keywords:** thrombosis, thrombolysis, fibrin, blood clot, isopeptide bond, leech, destabilase, factor XIII, thrombolytic drug

## Abstract

Leeches are amazing animals that can be classified as conditionally poisonous animals since the salivary cocktail they produce is injected directly into the victim, and its components have strictly defined biological purposes, such as preventing blood clot formation. Thrombolytic drugs are mainly aimed at treating newly formed blood clots. Aged clots are stabilized by a large number of isopeptide bonds that prevent the action of thrombolytics. These bonds are destroyed by destabilase, an enzyme of the leech’s salivary glands. Here, we conducted a pilot study to evaluate the feasibility and effectiveness of the use of destabilase in relation to blood clots formed during real pathological processes. We evaluated the isopeptidase activity of destabilase during the formation of a stabilized fibrin clot. We showed that destabilase does not affect the internal and external coagulation cascades. We calculated the dose–response curve and tested the ability of destabilase to destroy isopeptide bonds in natural blood clots. The effect of aged and fresh clots dissolving ability after treatment with destabilase coincided with the morphological characteristics of clots during surgery. Thus, recombinant destabilase can be considered as a potential drug for the treatment of aged clots, which are difficult to treat with known thrombolytics.

## 1. Introduction

Cardiovascular diseases are the leading cause of death worldwide. At the same time, a significant percentage of damage to the heart and main and peripheral vessels is associated with hypercoagulation and thrombosis, as well as their complications: myocardial infarction, stroke, and thromboembolism [1,2]. The thrombolytic drugs used in clinical practice include various kinds of modified serine proteinases related to the components of the blood fibrinolytic system [3], bacterial proteases [4,5], or metalloproteases from snake venom [6]. The mechanism of their action is to catalyze the proteolytic degradation of polypeptide bonds in the fibrin polymer molecules, which form the basis of a blood clot. Currently, four generations of such drugs have been distinguished. Modern thrombolytics surpass previous thrombolytics, with increased half-life and improved selectivity [7,8,9,10,11]. A significant drawback limiting the prescription of existing thrombolytics is the fact that the effectiveness of all four generations of drugs is determined by clot age: the older the clot, the more difficult it is to lyse it, as its structure changes over time [12,13]. Fibrin becomes stabilized by factor XIIIa due to the formation of strong isopeptide bonds that prevent the penetration of thrombolytic drugs into the clot [14]. It has been shown that isopeptide bonds formed between fibrin and α2-antiplasmin prevent thrombolysis [15]. 

Most thrombolytics used in clinical practice are aimed at lysing freshly formed blood clots [16]. The treatment of aged blood clots occurs most often through prolonged usage of direct oral anticoagulants, surgical treatment, or prophylaxis with venous cava filters [17]. There are currently no thrombolytic drugs that destroy isopeptide bonds in aged clots. However, research is currently underway to develop selective inhibitors of human factor XIIIa [18]. To date, very few studies have been conducted using factor XIII inhibitors [19,20], although direct inhibitors of coagulation factors are very clinically attractive. This may be due to the fact that transglutaminases are generally difficult to reach as a drug target [21]. Alternatively, one of the possible methods of thrombolytic therapy may be the effect on the factor’s XIII target, rather than its inhibition. Over the past several decades, research on the natural components of biological objects such as snake venom [22,23], bee venom [24,25], and leech saliva [26,27] have provided not only new tools for deciphering the molecular details of various physiological processes but also inspired the development and development of a number of therapeutic agents. In 1985, the ability of medicinal leech salivary gland cell secretion to dissolve stabilized, cross-linked with isopeptide ε-(γ-Glu) -Lys-bonds of fibrin, was discovered [28]. The secretion contains a protein called destabilase that destroys covalent bonds formed between fibrin monomers under the influence of factor XIIIa in blood plasma. Further studies showed that destabilase is an enzyme belonging to the i-type lysozymes group [29]. Destabilase consists of 115 amino acid residues and combines muramidase (EC 3.2.1.17) and endo-ε- (γ-Glu) -Lys-isopeptidase (EC 3.5.1.44) activity and antibacterial activity independent of the muramidase activity. A family of genes encoding destabilase has been identified [30,31]. Furthermore, all three known isoforms of destabilase from medicinal leeches were expressed in *E. coli* in the form of recombinant proteins [32]. Then, the second isoform of recombinant destabilase was obtained in human cells and characterized [33]. We described the effect of destabilase on stabilized fibrin and the product of its limited proteolysis, the D-dimer, the monomers of which are linked by isopeptide bonds [34]. However, the effect of recombinant destabilase on aged arterial and venous thrombi remains unclear.

In our previous study, we demonstrated the ability of recombinant destabilase to irreversibly lyse aged arterial thrombi in vivo in an experimental rat thrombosis model [35]. Here, we examined the mechanism of thrombolysis in vitro. We report that blood clots removed during surgical treatment of patients dissolved by isopeptidase activity of recombinant destabilase. Our approach is unique because it allows us to evaluate the direct effect of a thrombolytic agent not only on the size of a clot but also on the degree of its stabilization after treatment with destabilase.

## 2. Materials and Methods

### 2.1. Recombinant Destabilase Isolation

The isolation and purification of recombinant destabilase were performed as described previously [33]. Briefly, for protein production, the Expi293F cell line was transfected with a plasmid (pcDNA3.4-Dest2), expressing recombinant destabilase in the culture medium. Destabilase containing a C-terminal His-tag was purified using affinity metal-chelate chromatography using Ni-Sepharose (GE Healthcare, Chicago, IL, USA) with elution buffer containing 20 mM Tris, 500 mM NaCl, and 500 mM imidazole, pH 7.5. The isolated recombinant protein was analyzed qualitatively by SDS-PAGE. Protein concentration was measured using the Bradford method using Quick Start Bradford 1x Dye Reagent (Bio-Rad, Hercules, CA, USA). The measurement was performed using a MultiskanAscent scanner (Thermo Fisher Scientific, Waltham, MA, USA) with a light filter with a 695 nm threshold. 

### 2.2. Isopeptidase Assay

The assay was carried out using the Factor XIII-test kit (Renam, Moscow, Russia). The control plasma (Plasma-N, Renam) was recovered with distilled water to 90% of the required volume, and then destabilase was added up to the final volume to obtain a normal plasma concentration. A series of 2-fold dilutions of plasma was prepared with the dilution buffer (0.05 M imidazole, pH 7.4); then, a fibrinogen solution, kaolin suspension, thrombin–calcium mixture (human thrombin dissolved in 0.1 M calcium chloride) were added following the manufacturer’s instructions. The mixture was incubated for 30 min at 37 °C, after which 5% monochloroacetic acid was added (to render unstabilized fibrin soluble) [36], and the mixture was incubated for another 5 min at 37 °C. After incubation, the tubes were shaken, centrifuged, and the absorption of soluble protein in the supernatant was measured at 280 nm using a Cary50 spectrophotometer (Varian, Palo Alto, CA, USA).

### 2.3. Coagulation Tests

For routine coagulation tests, the APG4-03-Ph hematology analyzer (EMCO, Moscow, Russia), as well as MLT-APTT kit for activated partial thromboplastin time (APTT) determination, MLT-Thromboplastin for prothrombin time (PT) determination (MLT, Dubna, Moscow region, Russia), and thrombin reagent for thrombin time (TT) determination (Renam, Moscow, Russia), was used. All measurements were performed using control plasma.

### 2.4. Blood Clot Preparation

Blood clot fragments obtained during thrombectomy, thromboembolectomy, and thromboendarterectomy after atherosclerotic lesion and local thrombosis of various veins and arteries were collected. Patients were operated on urgently for acute vein thrombosis with signs of floating thrombus or acute ischemia resulting from:

1. Thromboembolism;
2. Acute thrombosis against the background of atherosclerotic artery stenosis, including hemorrhage into an atherosclerotic plaque;
3. Progression of previously existing subclinical arterial thrombosis.

Exclusion criteria: diabetes mellitus, different neoplasms, severe hepatic or renal failure, autoimmune disease, other acute diseases (including acute myocardial infarction, acute cerebrovascular accident), taking anticoagulants and antiplatelet agents within a month before this surgery, previous thrombotic therapy, absence of the need for emergency surgery for the underlying disease.

In total, 15 different blood clots of different localizations and ages were obtained from different patients (Table 1). 

All manipulations with patients and clinical material were approved by the hospital-based ethics committee. After intraoperative extraction of the clinical material, the material was washed and dehydrated overnight in a ScanVac CoolSafe lyophilizer (Labogene, Lillerød, Denmark) at 0.14 mbar. For further experiments, blood clots were divided into several 10–30 mg fragments, placed in 2 mL tubes, and weighed.

### 2.5. Thrombolysis by Destabilase and Streptokinase

Pre-weighted clot fragments were incubated in 2 mL tubes for 24 h either with the recombinant destabilase (0.4 mg/mL), streptokinase (units/mL) (Belmedpreparaty, Minsk, Belarus), or control buffer (20 mM Tris, 500 mM NaCl, 500 mM imidazole, pH 7.5). The volume of the solution was 20 mL per 1 g of the blood clot. Incubation was carried out in a shaker incubator at 37 °C at 150 rpm on a platform with a 20 mm rotation amplitude. Then, the blood clots were carefully washed with distilled water, maintaining the integrity of the blood clot, lyophilized overnight, and weighed. To determine the degree of clot lysis, the change in thrombus mass relative to the initial mass was determined.

### 2.6. Blood Clot Destabilization

We performed clot solubility assay, based on the ability of the unstabilized fibrin to dissolve in acetic acid or urea [37]. For this, 2% acetic acid was added to dehydrated blood clots previously treated with destabilase, streptokinase, or a control. The volume of acetic acid was 20 mL per 1 g of dry clot after treatment with the test solutions. Incubation was carried out in a shaker incubator at 37 °C at 150 rpm on a platform with a 20 mm rotation amplitude. After incubation, the samples were centrifuged, and the absorbance of the supernatant containing soluble fibrin was determined at a wavelength of 280 nm. 

### 2.7. Western Blotting 

Blood clots dissolved in 2% acetic acid diluted in Laemmli sample buffer were heated to 95 °C for 10 min. The probes were separated by 12% SDS-PAGE. Proteins were transferred (semi-dry transfer, 1 mA/cm^2^ for 1 h) to a 0.45 μm PVDF membrane (GE Healthcare). The membrane was blocked with 1% BSA, incubated with anti-fibrinogen antibody at a 1:400 dilution (Dako, Carpinteria, CA, USA) at 4 °C overnight, and then incubated with a horseradish peroxidase-linked secondary anti-rabbit IgG antibody (GE Healthcare) for 1 h. The membrane was visualized with ECL Plus Western blotting detection reagents (GE Healthcare) according to the manufacturer’s instructions. The signals were detected with the ChemiDoc XRS System (Bio-Rad, Hercules, CA, USA).

### 2.8. Statistics

Statistical analysis was performed using Prism 8 software (GraphPad, San Diego, CA, USA). All experiments were performed in three replicates. Mean values, standard deviations, and confidence intervals (CI95) were calculated for all data groups. To compare three independent groups for one characteristic, the ANOVA criterion according to Kruskal–Wallis was used. The critical significance level of the null statistical hypothesis was taken as 0.05.

## 3. Results

### 3.1. Isopeptidase Assay

**Figure 1:**
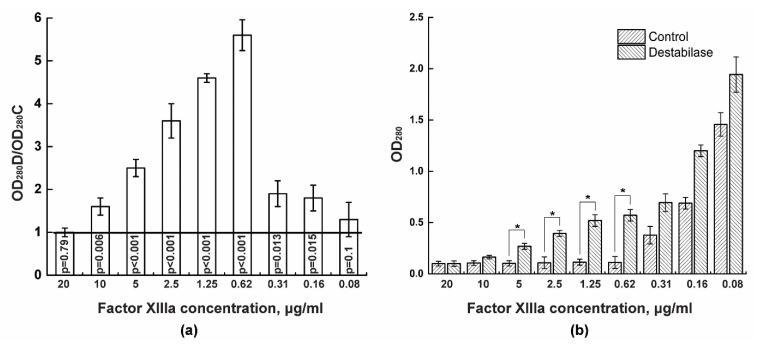
Determination of isopeptidase activity by the degree of dissolution of the fibrin clot in 5% monochloroacetic acid at various concentrations of factor XIII. Destabilase (10 μg/mL) was added to the plasma with a known concentration of factor XIII; then, two-fold plasma dilutions were obtained, and a fibrinogen with additional components was mixed to form a fibrin clot. After incubation, the fibrin clot was dissolved in 5% monochloroacetic acid, and the absorbance of the supernatant was determined at 280 nm. The optical densities of the samples with destabilase (OD_280_D) and control samples (OD_280_C) were compared as a ratio. The black line shows the threshold of the difference from the control. The *p*-values were calculated and placed in columns (**a**). The dependence of the OD_280_ of the dissolved fibrin clot degradation products on the factor XIIIa concentration in the presence or absence of destabilase was also compared. Differences were significant at *p* < 0.05 (*) (**b**).

We determined isopeptidase activity of recombinant destabilase by monitoring the ability of the unstabilized fibrin to dissolve in the 5% monochloroacetic acid. This assay shows the concentration of factor XIII at which a stabilized fibrin clot does not form and dissolves in monochloroacetic acid. We measured OD_280_ values for various dilutions of destabilase-containing plasma and control plasma and calculated their ratio. Samples in which destabilase prevented the fibrin clot stabilization, ratios greater than one, were obtained. Thus, it was shown that the addition of 10 μg/mL destabilase to plasma with 10 μg/mL factor XIII does not lead to stabilized fibrin clot formation with significance level *p* = 0.006 (Figure 1a). At the same time, in the control, an unstabilized fibrin clot remained in the test tube with 0.3125 μg/mL factor XIII. When comparing the absolute OD_280_ values, it can be seen that destabilase promotes the dissolution of fibrin clots already at a 10 μg/mL factor XIIIa concentration (Figure 1b). 

Then, we used a series of four-fold dilutions of destabilase and repeated the experiment to determine the minimum concentration that could destabilize the forming fibrin clot. As a result, destabilase at concentrations of 40, 10, 2.5, and 0.6 μg/mL significantly prevented the formation of a stabilized fibrin clot (Figure S1).

### 3.2. Coagulation Tests

We performed routine coagulation tests for activated partial thromboplastin time (APTT), prothrombin time (PT), and thrombin time (TT) to determine the effect of destabilase on internal and external blood coagulation cascades. These tests measure the time elapsed from activation of the coagulation cascade at different points to the generation of fibrin. For coagulation tests, 40 μg/mL recombinant destabilase was used. As a result, no significant differences were found between the control plasma and plasma with destabilase in coagulation tests (Table 2). 

### 3.3. Thrombolysis by Destabilase or Streptokinase

To assess the thrombolysis caused by destabilase (isopeptidolysis) and streptokinase (proteolysis), we compared the blood clots’ weight change before and after treatment with the drugs. The extracted blood clots were characterized by time (aged/fresh) and localization (arterial/venous vessels), based on clinical data and the morphology of the clots (Table 1). Blood clots older than 48 h were considered to be aged [38]. All extracted blood clots were lyophilized and weighted. The ratio of the dry weight of treated blood clots to the dry weight of native blood clots, expressed as a percentage was determined. In the experiment, weight decrement was noted for all samples. At the same time, the samples treated with the control buffer showed a maximum change in weight by 7% and the samples treated with destabilase and streptokinase by 31% and 29%, respectively. It is noteworthy that those blood clot fragments that lost more weight during treatment with destabilase lost less weight during treatment with streptokinase and vice versa (Figure S2). 

### 3.4. Blood Clot Destabilization

**Figure 2:**
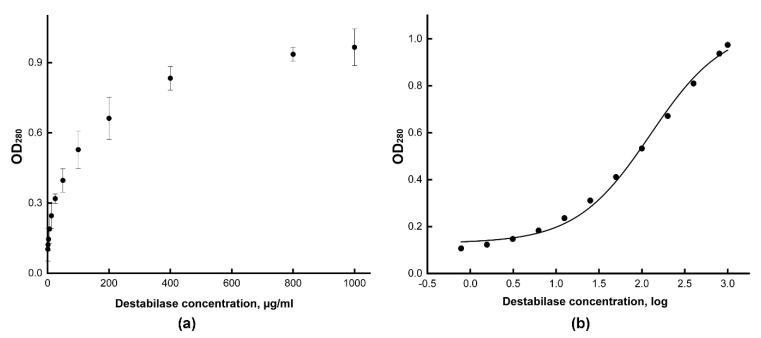
Blood clot destabilization with various destabilase concentrations. We measured the optical density at 280 nm of a clot products solution after dissolution in 2% acetic acid. The clots were pretreated with various destabilase concentrations from 0.7 µg/mL to 1000 µg/mL. The OD_280_ values are expressed as mean ± standard deviation (**a**). To determine the working concentration of destabilase, the dose–response sigmoid curve was plotted (**b**). The EC_50_ and EC_80_ concentrations were defined.

To analyze the destabilization of residual thrombi after treatment with destabilase, we dissolved them in acetic acid to obtain soluble unstabilized fibrin and measured the absorption of the resulting solution. At the first stage, we determined the optimal concentration of recombinant destabilase by treating blood clot fragments with various destabilase concentrations. We obtained the dependence of the optical density (OD_280_) of a dissolved clot solution in 2% acetic acid on the destabilase concentration (Figure 2a). Next, we obtained the logarithmic values of the destabilase concentration, plotted a sigmoid dose–response curve, and determined the values of the half-maximal effective concentration (EC_50)_ and 80% maximal response concentration (EC_80_) for the recombinant destabilase (Figure 2b). The EC_50_ value corresponds to 115 µg/mL, and the EC_80_ corresponds to 405 µg/mL recombinant destabilase concentration. The working concentration for further experiments was rounded up to 400 µg/mL.

**Figure 3:**
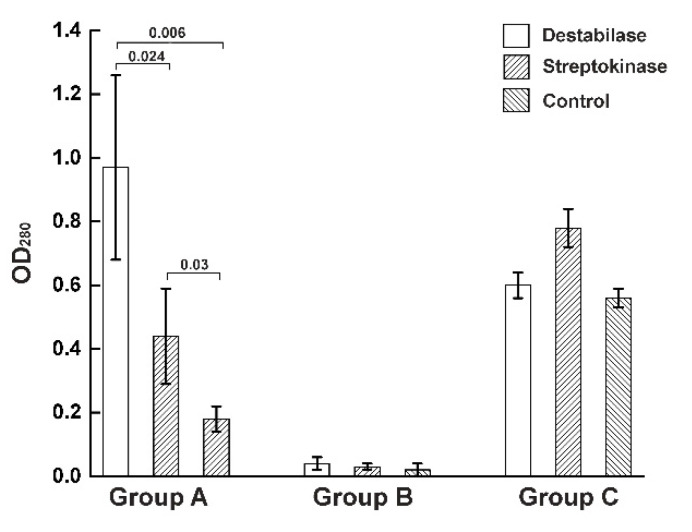
Clustering data analysis of the degree of destabilization of blood clots. Three groups were formed: A, a group of blood clots that dissolve better in 2% acetic acid after treatment with destabilase than after treatment with streptokinase or control buffer; B, a group of blood clots that do not dissolve in acetic acid; C, a group of blood clots that dissolve well in acetic acid, regardless of the solution with which the samples were treated. The OD_280_ axis shows the absorbance of the supernatant of a dissolved in 2% acetic acid blood clots at a wavelength of 280 nm. Each group contains mean OD_280_ values of samples after blood clot destabilization assay and data clustering. The data are expressed as mean ± CI95. Significant differences are indicated by asterisk brackets with the *p*-value. The significance level of the null statistical hypothesis was taken as 0.05.

Next, all dried samples treated with destabilase, streptokinase, and control from a previous assay were incubated in 2% acetic acid for 24 h. The optical densities of the dissolved clot solution supernatants were measured at a wavelength of 280 nm, and the values were divided into three groups based on the efficacy of the destabilase using the Online Hierarchical Clustering calculator [39] (Figure S3). Samples that dissolved better in acetic acid after destabilase treatment than after treatment with streptokinase or control buffer were assigned to Group A. Then, blood clots that did not dissolve in acetic acid were assigned to Group B. Blood clots that dissolved in acetic acid, regardless of the solution used, were assigned to Group C (Figure 3 and Figure S4). 

**Figure 4:**
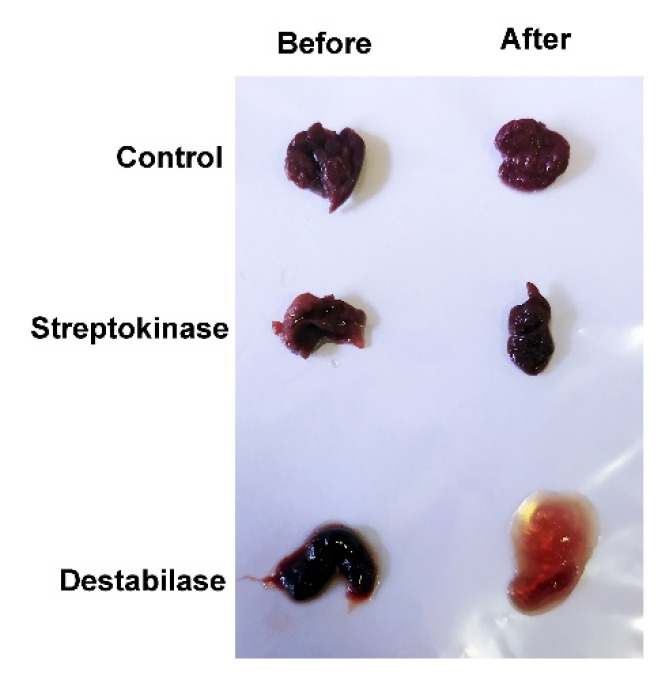
Blood clot morphology change photograph. The dry blood clot was divided into parts, incubated with destabilase (400 µg/mL), streptokinase (U/mL), and control buffer for 24 h, and lyophilized. Then, three parts were incubated in 2% acetic acid solution for the next 24 h, and additional three parts were rehydrated in 2% acetic acid for 10 min prior to taking a photograph.

It was shown that all aged blood clots used in the experiment clustered into Group A. The absorption level of the samples treated with destabilase (0.96 ± 0.27) was significantly different from both the samples treated with streptokinase (0.48 ± 0.13) at *p* = 0.0236 and with the control buffer (0, 17 ± 0.03) at *p* = 0.006. At the same time, in Groups B and C, no significant differences were found between the samples. It was also shown that the morphology of aged blood clots treated with destabilase changed (Figure 4). 

### 3.5. Fibrinolytic Activity of the Destabilase

**Figure 5:**
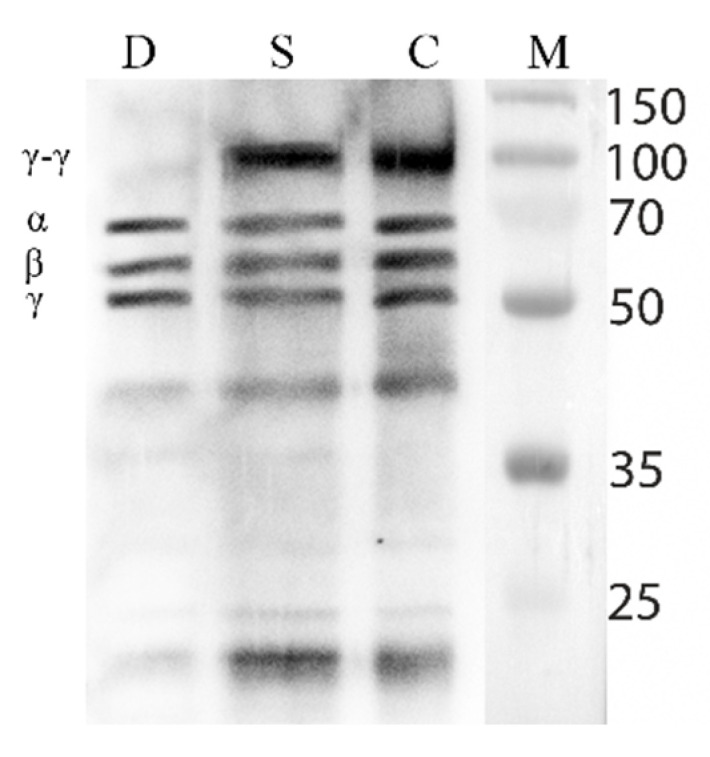
Fibrinolytic activity of destabilase. The Western blot with polyclonal anti-fibrinogen antibody of blood clot fragments treated with destabilase (D), streptokinase (S), and control buffer (C). M—pre-stained protein ladder (15–150 kDa), α, β, γ—α, β, γ-chains of human fibrin (63.5, 56, and 47 kDa, respectively).

To visualize the result of the fibrinolytic activity of the destabilase, we performed Western blot assay of the blood clots dissolved in 2% acetic acid. We analyzed blood clots treated with destabilase, streptokinase, and control buffer solution (Figure 5). 

## 4. Discussion

In 1991, Baskova described a fundamentally new mechanism of thrombolysis, called “isopeptidolysis” [40], which consists in catalyzing the destruction of isopeptide bonds formed in the process of fibrin stabilization. This process is activated by destabilase, an enzyme, secreted from the salivary glands of a medicinal leech. It was suggested that destabilase better destroys aged blood clots, rather than those newly formed. In this study, we investigated the ability of recombinant destabilase to lyse aged blood clots extracted from patients. 

Destabilase hydrolyzes endo-ε- (γ-Glu) -Lys-isopeptide bonds, which are formed between monomers of γ-γ fibrin chains in blood clots, that are formed due to factor XIIIa. It was previously shown that α-(α-Glu)-Lys substrate remains unchanged during treatment with destabilase [41]. Highly cross-linked blood clots are known to be less sensitive to thrombolysis [42]. 

Previously, we isolated and characterized the recombinant destabilase of a medicinal leech. The isopeptidase activity of the destabilase, based on the cleavage of the chromogenic substrate L-γ-Glu-pNA, and fibrinolytic activity, based on a modified test with fibrin plates, were demonstrated [32]. In this study, for the first time, we showed the isopeptidase activity of recombinant destabilase in the presence of factor XIII in plasma. In this test, factor XIII is converted to factor XIIIa at the final stage of the coagulation cascade under the action of thrombin in the presence of Ca^2+^ ions. As a result, ε- (γ) -glutamyl-lysine bonds between fibrin monomers form. In this study, we showed that by adding 40 to 0.6 μg/mL destabilase to control plasma, the fibrin clot begins to dissolve in the presence of 10 μg/mL factor XIII. In control plasma, the fibrin clot begins to dissolve only in the presence of 0.3 μg/mL factor XIII (Figure 1 and Figure S2). It is known that this method is used to diagnose factor XIII deficiency [43]. Thus, while factor XIIIa is active during thrombus aging, destabilase can destroy formed isopeptide bonds. 

Coagulation tests are used to identify hypo- and hypercoagulation, including deficiency of factors VIII, IX, X, and XI (intrinsic pathway), factors of the prothrombin complex, and fibrinogen–fibrin conversion [44]. In APTT, TT, and PT coagulation tests, we demonstrated the absence of significant differences between plasma with destabilase and control plasma (Table 2). One of the most serious side effects of thrombolytic drugs is bleeding due to their effect on various coagulation factors [45]. Thus, destabilase does not affect the coagulation factors of the intrinsic and extrinsic pathways, as it has no proteolytic action. It acts only at a stabilized blood clot. 

We performed the pilot study to determine an appropriate sample size for the full-scale project and to evaluate the feasibility and effectiveness of the use of destabilase in relation to blood clots formed during real pathological processes. Here, we used 15 blood clots samples extracted from 12 patients during surgery (Table 1). All blood clot fragments were characterized. When determining the temporal characteristics, in addition to the clinic and the history of the disease, we focused on the following characteristics of the studied formations:

1. Fresh clots—dark, easily detached from the vessel wall and fragmented when pressed with fingers;
2. Aged clots—light, fixed to the vessel wall, dense, fragmented only with a sharp instrument;
3. Aged embolus—a denser and lighter fragment of an aged intracardiac clot (its presence was confirmed by transesophageal ultrasound in the postoperative period),
4. Atherosclerotic plaque—located subintimal, heterogeneous, can be calcified.

To characterize thrombolysis of the clots, we compared the dry weight of a blood clot before and after treatment with destabilase and streptokinase. We chose streptokinase as a comparison drug since it is a good thrombolytic agent and, in some cases, is used as an emergency drug [46]. The thrombolysis was studied, and a change in the weight of blood clots was found in all groups: blood clots treated with destabilase, streptokinase, and control buffer. Figure S2 shows that blood clots treated with a buffer were characterized by the least weight loss. This may be due to the mechanical leaching of blood components that were not leached during extraction. Streptokinase is a systemic thrombolytic drug that promotes activation of the cascade of proteins of the blood fibrinolytic system for plasminogen–plasmin conversion [47,48]. Streptokinase, similar to other thrombolytic drugs such as recombinant tissue plasminogen activator, is most effective in treating fresh blood clots. Thus, its use is most effective in the first 3–6 h [49]. Aged blood clots are not lysed by streptokinase because these blood clots contain a large number of isopeptide bonds. Residue Gln397 of the gamma chain of one fibrin monomer molecule and residue Lys405 of the gamma chain of another molecule are involved in the formation of isopeptide bonds. After the destruction of isopeptide bonds, a modified unstabilized fibrin polymer is formed, in which Glu and Lys residues are present instead of the initial Gln and Lys residues. The appearance of Glu instead of Gln is critical because it leads to spontaneous depolymerization of the modified unstabilized fibrin polymer, which was demonstrated in an in vitro study as the appearance of liquid zones of the modified fibrin monomer on fibrin plates [34]. Thus, aged blood clots respond better to thrombolysis after the action of destabilase than after the action of streptokinase. 

At the same time, samples from blood clots in an atherosclerotic plaque did not dissolve from the action of destabilase or from the action of streptokinase. Atherosclerotic plaque is a dense lipid-calcified structure that is not only morphologically very different from fresh and aged blood clots but also not vulnerable to thrombolysis [50]. 

Here, for the first time, we performed a destabilization analysis of blood clots extracted from patients. It is based on the solubility of unstabilized fibrin in 2% acetic acid, as it is known that under these conditions only unstabilized fibrin dissolves [51,52]. We clustered blood clot samples based on their ability to be dissolved after pretreatment with destabilase (Figures S3 and S4). As a result, we divided the blood clots into three groups. One feature of aged blood clots is the inability of aged clots to be dissolved in 2% acetic acid, as shown in Figure 3 (Figure 3, Group A). The blood clots in Group C dissolved in 2% acetic acid, regardless of the treatment with any drug (Figure 3, Group B). This suggests that these blood clots are fresh enough and do not have enough cross-linking for stability in acetic acid. Blood clots older than 48 h are known to be aged and poorly lysed by streptokinase [38]. Group B included thrombi in an atherosclerotic plaque that did not dissolve in acetic acid due to its morphology. As shown in Figure 3, the confidence interval was largely scattered since the blood clots of the same age from different patients may differ, and the structure may be heterogeneous inside one blood clot [53]. In this study, there were three patients from whom both aged and fresh blood clots were extracted, and they were clustered into different groups not only morphologically but also after measuring the thrombolytic activity of destabilase. For aged clots treated with destabilase, a change in morphology (Figure 4) was noticed that was not observed in other cases. The results were supported by Western blot assay (Figure 5) that demonstrated the isopeptidase activity of the enzyme by degradation of the γ-γ fibrin chain. These data correlate with previously obtained results of measuring the diameters of the lysed zones that resulted from the placement of droplets of the enzyme-containing solutions on fibrin plates with subsequent SDS-PAGE analysis [32].

Destabilase acts not like emergency thrombolytic drugs; it has a slow effect [40]. Previously, it was shown that after intravenous administration to rats after 67 h, the experimentally obtained venous thrombi were lysed only by 60–70%, and complete lysis was observed only after 137 h [54]. A low rate of thrombolysis is a positive property of destabilase since it correlates with a low repair rate of the damaged vascular wall, a factor that potentially prevents the recurrence of the pathological process due to the formation and fixation of a clot in the area of the injured endothelium [55]. Furthermore, some thrombolytic agents that induce the formation of unstabilized fibrin by degradation XIII and XIIIa factors may cause bleeding [56]. Destabilase does not demonstrate such side effects, as it has no proteolytic effect and does not affect the degradation of XIII and XIIIa factors [57]. This property may be used for developing new thrombolytic drugs targeting aged blood clots. 

Based on the obtained results, it is clear that destabilase promotes thrombolysis of aged blood clots, converting stabilized fibrin to unstabilized fibrin by isopeptidolysis. The result is a blood clot that can be lysed under the action of plasminogen activators. Destabilase disruption of isopeptide bonds in stabilized aged blood clots is a new type of proteolysis-independent thrombolysis that can be supplemented by the introduction of classical thrombolytic drugs that activate plasminogen.

## Figures and Tables

**Table 1:** Characterization of the extracted blood clots used in the study.

Sample	Age	Characteristics	Diagnosis	Comorbidity
T1	63	An aged clot fragment from the right femoral artery	ASVD. Aortic, iliac, left femoral, and popliteal arteries stenosis with thrombotic occlusion of the right femoral and popliteal arteries.	IHD, old myocardial infarction
T2	63	A fresh right femoral arterial clot fragment *	ASVD. Aortic, iliac, left femoral, and popliteal arteries stenosis with thrombotic occlusion of the right femoral and popliteal arteries.	IHD, old myocardial infarction
T3	68	A fresh right femoral arterial clot fragment *	ASVD of the femoral, popliteal, and tibial arteries. Right femoral artery thrombosis	IHD, HT
T4	76	A mixed thromboembolism from the left brachial artery (old embolus and fresh continued clot) *	Left brachial artery thrombosis	IHD, Permanent A-fib, HT
T5	74	An aged right popliteal clot fragment	ASVD. Aortic, iliac, femoral, tibial, and left popliteal arteries stenosis with thrombotic occlusion of the right popliteal artery	IHD, chronic cholecystitis, chronic pancreatitis, chronic bronchitis.
T6	75	An aged femoral arterial clot fragment from the proximal part of the left femoral artery, intimately associated with the atherosclerotic plaque	ASVD. Aortic, iliac, popliteal, tibial, and right femoral arteries stenosis with thrombotic occlusion of the left femoral artery.	IHD, megaloblastic anemia, chronic gastritis, hip, and knee osteoarthritis
T7	75	An aged femoral arterial clot fragment from the distal part of the left femoral artery, intimately associated with the atherosclerotic plaque	ASVD. Aortic, iliac, popliteal, tibial, and right femoral arteries stenosis with thrombotic occlusion of the left femoral artery.	IHD, megaloblastic anemia, chronic gastritis, hip, and knee osteoarthritis
T8	65	The fresh blood clot fragment up to 8 cm long in the right tibial vein, floating in the popliteal vein. During surgical treatment for an open fracture, thrombectomy was performed *	Open comminuted fractures of the tibia and fibula with displacement. Floating clot of the right tibial and popliteal veins.	Post-traumatic bleeding, HT
T9	74	An aged left femoral arterial clot fragment	ASVD. Iliac, right femoral, and popliteal arteries, right posterior tibial artery stenosis with thrombotic occlusion of the left femoral and popliteal arteries.	HT
T10	70	Fragment of an organized aged clot fragment from the right brachial artery	Thromboembolism of the right brachial artery	IHD, Permanent A-fib
T11	78	A fresh left femoral arterial clot fragment *	ASVD. Aortic, iliac, right femoral, and popliteal arteries stenosis with thrombotic occlusion of the left femoral and popliteal arteries	IHD, HT, Right kidney cyst.
T12	72	A fresh right femoral arterial clot fragment *	ASVD. Tibial and left popliteal arteries stenosis with thrombotic occlusion of the right popliteal and femoral arteries.	IHD, COPD
T13	72	An aged right popliteal clot fragment	ASVD. Tibial and left popliteal arteries stenosis with thrombotic occlusion of the right popliteal and femoral arteries.	IHD, COPD
T14	66	The apex fragment of the floating part of the left femoral vein clot **	Occlusive thrombosis of the tibial and popliteal veins, floating thrombosis of the left femoral veins.	Injury: partial rupture of the Achilles tendon, IHD, HT, chronic cholecystitis, chronic pancreatitis.
T15	68	The fragment of fresh clot from the right brachial artery *	Thromboembolism of the right brachial artery	IHD, permanent A-fib, HT, chronic gastritis, chronic duodenitis, postcholecystectomy syndrome

Patients were operated on urgently for acute vein thrombosis with signs of embolism hazardous flotation or acute ischemia. All fragments of clots, emboli, and atherosclerotic plaques were forwarded to morphological study, and their fundamental characteristics were confirmed. In this study, all patients were men. ASVD—arteriosclerotic vascular disease, IHD—ischemic heart disease, HT—hypertension, COPD—chronic obstructive pulmonary disease, A-fib—atrial fibrillation, *—less than 3 h passed from the acute ischemia to thromboembolectomy, **—sonography #1: occlusive popliteal vein thrombosis, the patient refused from treatment; sonography #2: 13 days later: hypoechoic femoral vein floating clot up to 7 cm, the patient was operated 5 h after sonography #2: floating part up to 9 cm. T1/T2, T6/T7, T12/T13—a pair of fresh and aged clots from one patient.

**Table 2:** Coagulation tests results.

Test Name:	APTT	TT	PT
Destabilase	35 ± 2.3	20.2 ± 1.2	11.3 ± 0.8
Control	34.3 ± 1.5	19.5 ± 1.4	10.8 ± 0.4

For coagulation tests, the APG4-03-Ph coagulometer was used. All measurements were performed using normal plasma (Renam), and 40 μg/mL recombinant destabilase was used. Normal plasma with imidazole buffer was used as a control. All experiments were repeated five times. Data are expressed as mean time in seconds ± standard deviation. There were non-significant differences between samples.

## Supplementary Materials

The following are available online at https://www.mdpi.com/article/10.3390/cimb43030143/s1, Figure S1: Determination of isopeptidase activity by the degree of dissolution of the fibrin clot in 5% monochloroacetic acid in the presence of various concentrations of factor XIII, Figure S2: Determination of the degree of thrombolysis stimulated by streptokinase and destabilase, Figure S3: Cluster analysis of the blood clots, Figure S4: The degree of destabilization of blood clots.
