# Supplementary material for: Recombinant Destabilase from Hirudo medicinalis Is Able to Dissolve Human Blood Clots In Vitro
Source: Curr Issues Mol Biol. 2021 Nov 20;43(3):2068–81. doi: 10.3390/cimb43030143 (PMC8929072; doi:10.3390/cimb43030143)
Supplement: Supplementary file 1 [file cimb-43-00143-s001.zip › cimb-1442703-supplementary for conversion from author.pdf]

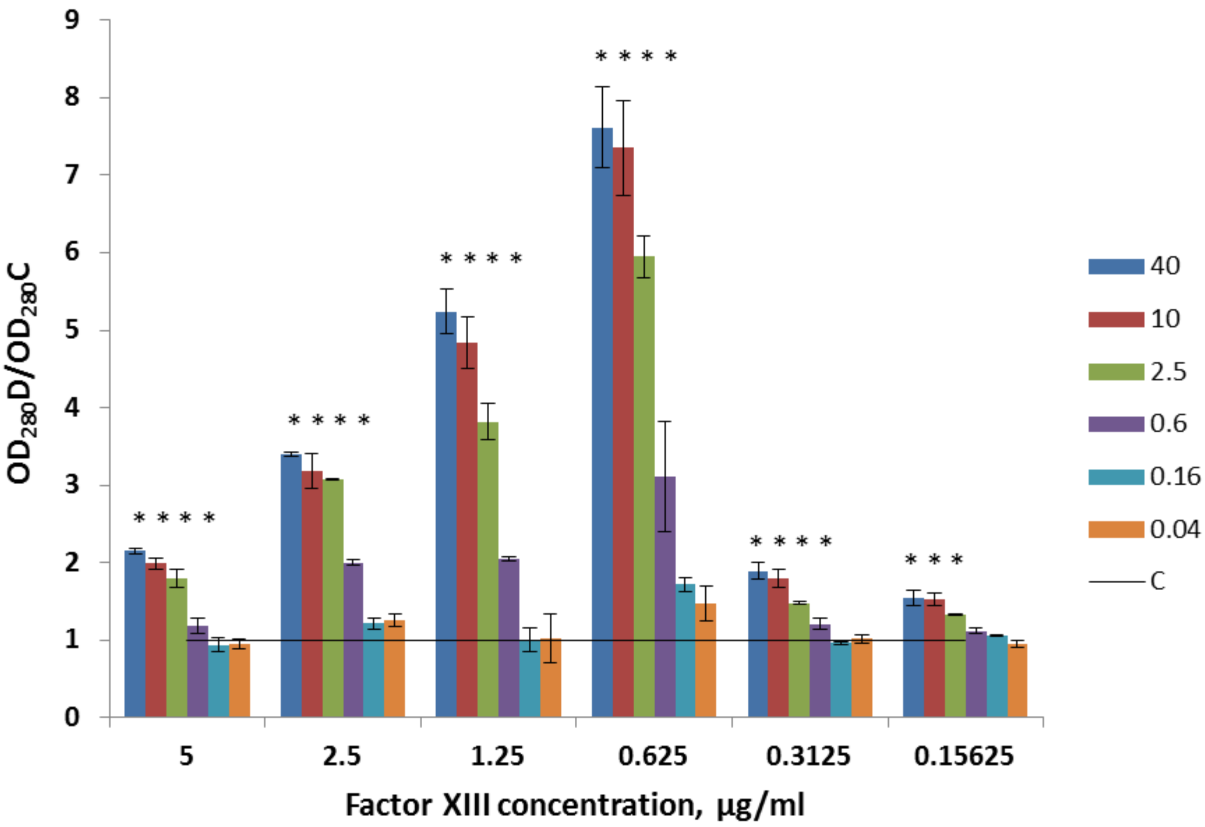

Figure S1. Determination of isopeptidase activity by the degree of dissolution of the fibrin clot in 5% monochloroacetic acid in the presence of various concentrations of factor XIII. Destabilase at different concentrations (40, 10, 2.5, 0.6, 0.16, and 0.04 µg/ml) was added to the plasma, after which two-fold plasma dilutions were obtained, and a kaolin, fibrinogen and a thrombin-calcium mixture was added to form a fibrin clot. After 30 minutes of incubation, the fibrin clot was dissolved in 5% monochloroacetic acid, the samples were centrifuged, and the absorbance of the supernatant was determined at 280 nm. Then, the optical densities of the samples with destabilase ($OD_{280}D$) and control samples ($OD_{280}C$) were compared as a ratio. The black line shows the threshold of the difference from the control. The black line shows the control/control ratio. Differences were significant at $p<0.05$ (*).

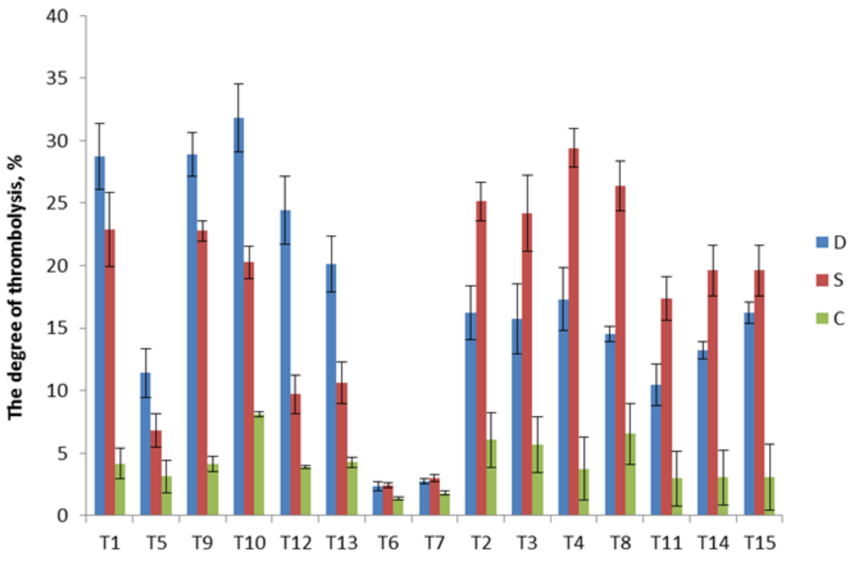

Figure S2. Determination of the degree of thrombolysis stimulated by streptokinase and destabilase. The degree of thrombolysis was determined as the change in the dry weight of the treated clot relative to the initial weight, expressed as a percentage. Thrombi were dehydrated, weighed, and treated with destabilase (D), streptokinase (S) and control buffer (C) for 24 hours. Then, the blood clots were washed, dried and weighed.

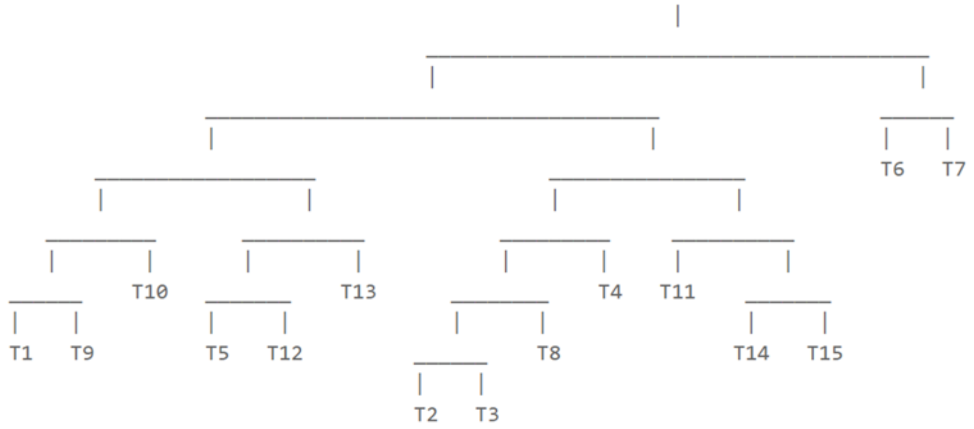

Figure S3. Cluster analysis of the blood clots. The extracted clots (T1-T15) were divided into parts and treated with destabilase, streptokinase, and control buffer. After treatment, clots were washed, dehydrated and incubated with 2% acetic acid. Then, the OD280 of the supernatants was measured. Cluster analysis was performed based on the ratio of the optical density of a clot sample pretreated with destabilase to that of a sample pretreated with streptokinase and a control.

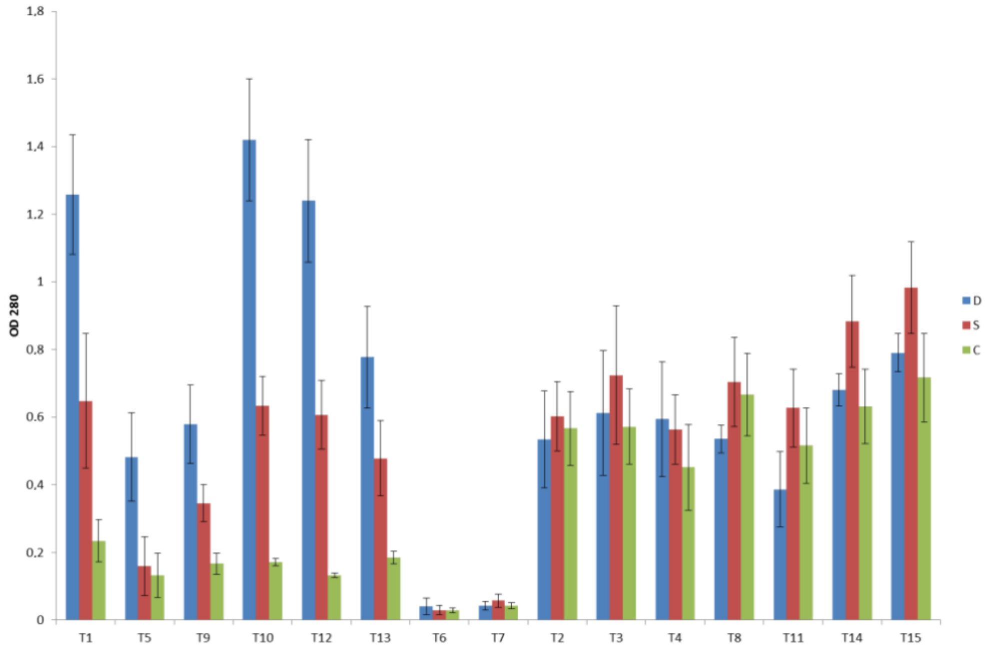

Figure S4. The degree of destabilization of blood clots. Blood clots were pretreated with destabilase (D), streptokinase (S) and control buffer (C). Then, they were incubated in 2% acetic acid and centrifuged, and the absorbance of the supernatant was determined at 280 nm. Blood clots were grouped as follows: blood clots that dissolved better in 2% acetic acid after treatment with destabilase than after treatment with streptokinase or control buffer (T1, T5, T9, T10, T12, and T13), blood clots that did not dissolve in acetic acid (T6 and T7), and blood clots that dissolved well in acetic acid, regardless of the solution with which the samples were treated (T2, T3, T4, T8, T11, T14, and T15).

Table S1. Characterization of the extracted blood clots used in the study.

SAMPLE	CHARACTERISTICS
**T1**	An aged femoral arterial clot
**T2**	A fresh femoral arterial clot
**T3**	A fresh femoral arterial clot
**T4**	A mixed clot from upper limb artery
**T5**	An aged popliteal clot
**T6**	An aged femoral arterial clot with the atherosclerotic plaque
**T7**	An aged femoral arterial clot with the atherosclerotic plaque
**T8**	A fresh venous clot from leg vein
**T9**	An aged femoral arterial clot
**T10**	An aged clot from upper limb artery
**T11**	A fresh femoral arterial clot
**T12**	A fresh femoral arterial clot
**T13**	An aged popliteal clot
**T14**	A fresh venous clot from leg vein
**T15**	A fresh clot from upper limb artery

Based on the clinical data and the appearance of the blood clot, a table was generated with the working names of blood clots (T1-T15).
